# Wearing an eye mask during overnight sleep improves episodic learning and alertness

**DOI:** 10.1093/sleep/zsac305

**Published:** 2022-12-15

**Authors:** Viviana Greco, Damiana Bergamo, Paola Cuoccio, Karen R Konkoly, Kike Muñoz Lombardo, Penelope A Lewis

**Affiliations:** Cardiff University Brain Research Imaging Centre (CUBRIC), School of Psychology, Cardiff University, Cardiff, UK; IMT School for Advanced Studies Lucca, Lucca, Italy; Department of Psychology, University of Padua, Padova, Italy; Department of Psychology, Northwestern University, Chicago, IL, USA; Cardiff University Brain Research Imaging Centre (CUBRIC), School of Psychology, Cardiff University, Cardiff, UK; Cardiff University Brain Research Imaging Centre (CUBRIC), School of Psychology, Cardiff University, Cardiff, UK

**Keywords:** sleep, eye mask, learning, episodic memory, alertness

## Abstract

Ambient light can influence sleep structure and timing. We explored how wearing an eye mask to block light during overnight sleep impacts memory and alertness, changes that could benefit everyday tasks like studying or driving. In Experiment 1, ninety-four 18–35-year-olds wore an eye mask while they slept every night for a week and underwent a control condition in which light was not blocked for another week. Five habituation nights were followed by a cognitive battery on the sixth and seventh days. This revealed superior episodic encoding and an improvement on alertness when using the mask. In Experiment 2, thirty-five 18–35-year-olds used a wearable device to monitor sleep with and without the mask. This replicated the encoding benefit and showed that it was predicted by time spent in slow-wave sleep. Our findings suggest that wearing an eye mask during overnight sleep can improve episodic encoding and alertness the next day.

## Graphical Abstract

**Graphical Abstract:**
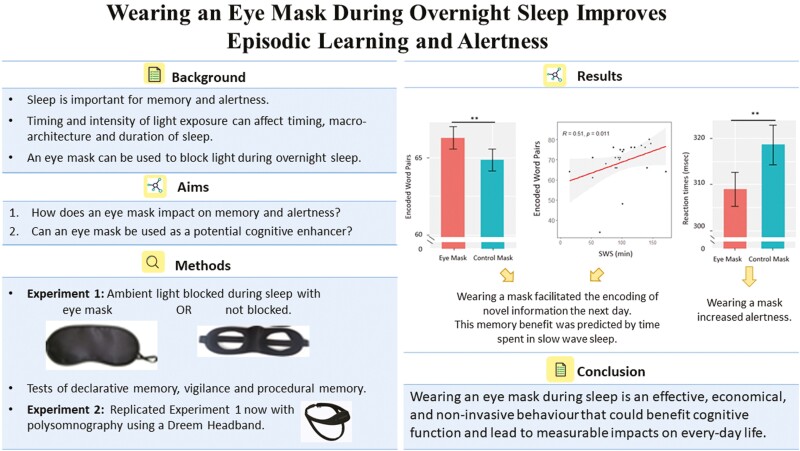

**Statement of significance**

Sleep is crucial for alertness and for preparing the human brain to encode new information. However, it can be disrupted by external stimuli such as light or sounds. This study explored wearing an eye mask as a potential cognitive enhancer which protects overnight sleep by blocking ambient light. We found that wearing a mask increased alertness and facilitated the encoding of novel information the next day. Furthermore, the benefit to memory was predicted by time spent in slow-wave sleep while wearing the mask. This suggests wearing an eye mask during sleep is an effective, economical, and noninvasive behavior that could benefit cognitive function and lead to measurable impacts on everyday life.

## Introduction

Sleep plays an essential role in many physiological functions including immune control, energy conservation, homeostatic restoration, and memory processing [1–3]. Sleep quality and quantity are crucial for brain function, and studies have indicated that a night of sleep deprivation or multiple nights of partial sleep restriction have a negative impact on cognition, for instance, affecting subsequent memory encoding [4–7]. Moreover, optimal academic performance is strongly related to the timing, quality, and quantity of sleep [8].

In mammals, the sleep–wake cycle is regulated by the suprachiasmatic nuclei (SCN) of the anterior hypothalamus [9]. SCN activity is strongly synchronized by the light–dark cycle via intrinsically photosensitive retinal ganglion cells [10, 11]. The tight interaction between light and sleep regulation is, therefore, clear, with a large body of evidence supporting the impact of light on sleep timing, macro-architecture, and duration [10, 12–16]. A recent study conducted by Wams et al. assessing the link between light exposure and subsequent sleep, revealed that subjects with earlier exposure to light spent significantly more time in slow-wave sleep (SWS) at the expense of rapid-eye movement (REM) sleep [10].

Non-pharmacological methods for improving sleep are a topic of great current interest [17], and the use of an eye mask to prevent light from reaching the retina during overnight sleep has been demonstrated to positively affect self-reported sleep quality in intensive care units where patients are systematically exposed to high levels of light [18, 19]. In the current study, we set out to investigate the benefits of wearing an eye mask to block light during normal sleep in the home. Declarative learning [4] and vigilant attention [7, 20] are both known to be sleep sensitive. Given the practical importance of these abilities in everyday life, for instance in studying at school or in driving a car [21], we wanted to examine the impact of eye mask manipulation of these abilities. With this aim in mind, we ran a within-subject design to look at these cognitive processes (Experiment 1) and a follow-up study that examined sleep architecture (Experiment 2).

## Methods

### Participants

All participants were healthy volunteers, with no history of drug/alcohol abuse, psychological, neurological, or sleep disorders. We selected participants who reported no hypersensitive skin or contact allergies and no problems falling asleep with open shutters and wearing both an eye mask and a wearable EEG device (Dreem headband, DH [22]). Participants agreed to abstain from alcohol and caffeine throughout the experiment. Additionally, the online screening ensured that they had not worn an eye mask for sleep before and they agreed not to nap on the days of the experiment.

The sample size of Experiment 1 was determined using a power calculation based on a pilot study (*n* = 8) on the Paired associate learning (PAL) task and based on a paired-sample *t*-test (G*Power Version 3.1.9.6) [23]. This pilot predicted 80% power to detect a medium-size effect (Cohen’s *d* = 0.3) with 88 subjects and a conventional α of 0.05. Ninety-four native English speakers (59F, age range: 18–35 years, *M* = 21.07, *SD* = 2.74) took part in the study. Of these, five were excluded due to voluntary withdrawal, so our final dataset included 89 participants (54F, *M* = 20.98, *SD* = 2.68). Due to technical failures when executing the tasks, a further six participants were excluded from the PAL, four were excluded from the Psychomotor Vigilance Test (PVT), and three from the motor-skill learning (MSL) analysis.

Experiment 2 was undertaken by 37 native English and Italian speakers (29F, age range: 18–35 years, *M* = 23.03, *SD* = 3.52). Of these, four were excluded due to difficulty falling asleep before 05:00 am (*n* = 1), sudden notification of working commitments (*n* = 1), and voluntary withdrawal (*n* = 2). Our final dataset therefore included 33 participants (25F, *M* = 23.09, *SD* = 3.57). Sample size was predetermined using a formal power analysis for correlation analysis. A sample size of 29 was needed to detect a correlation coefficient of 0.5 with a conventional *α* of 0.05 and 80% power. The number recruited slightly exceeded the number needed. All participants gave informed consent for the experiments, which were approved by the Ethics Committee of the School of Psychology at Cardiff University, and received monetary compensation for their participation.

### Procedure

**Figure 1.**
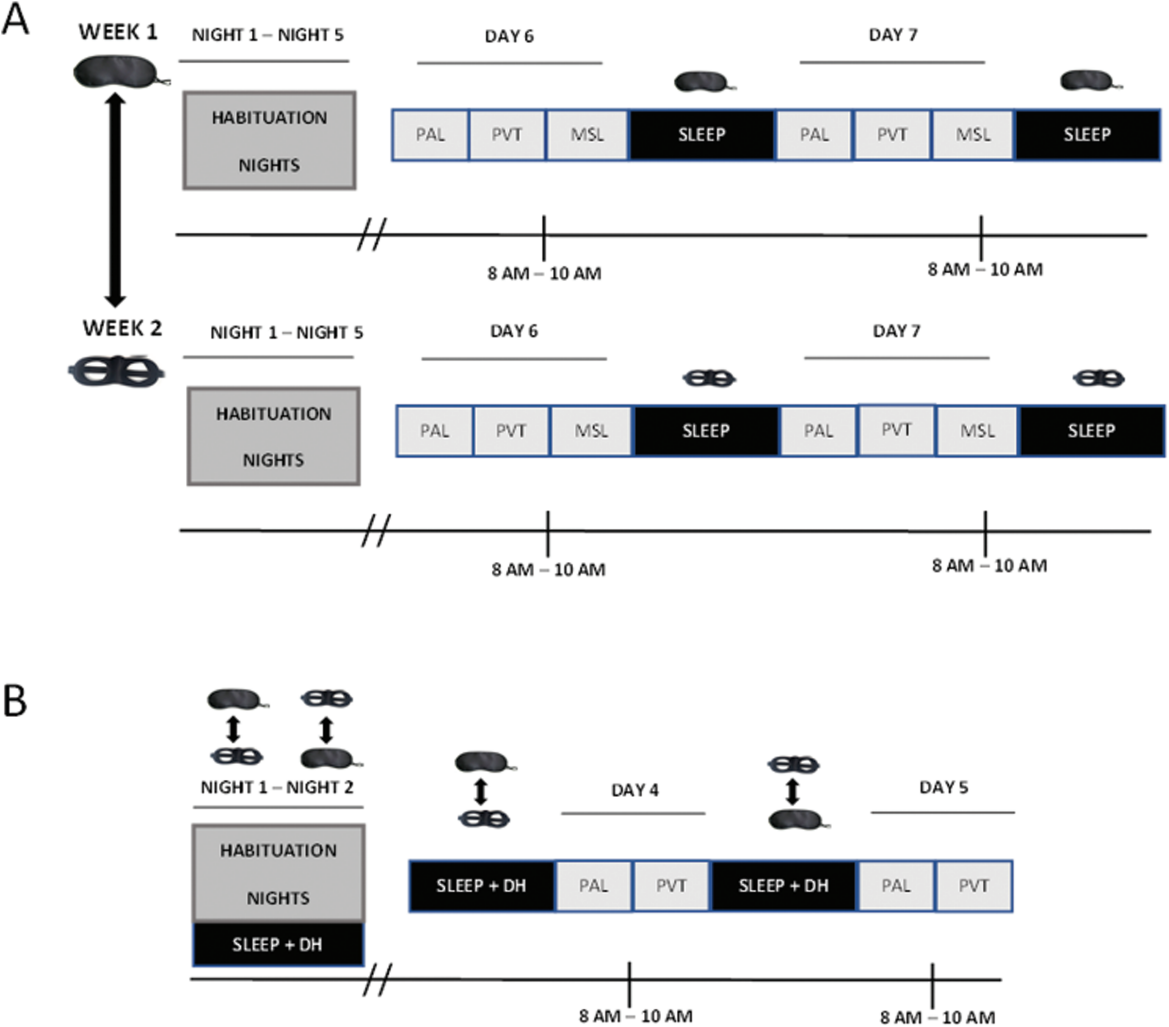
Experimental procedure. (A) Experiment 1 consisted of 2 consecutive weeks in which, in a counterbalanced order, ambient light was blocked with an eye mask during sleep for 1 week, or not blocked with a control mask for the other week. Night 1–Night 5: participants slept at home wearing a mask (eye mask or control). Day 6–Day 7: participants performed the PAL, the PVT, and the MSL task. (B) Experiment 2 consisted of 5 days, 2 habituation nights, and 2 experimental days. For the entire study duration, participants slept with an eye mask or a control mask (counterbalanced order) together with the DH. In the morning of Days 4 and 5, participants completed the PAL and the PVT.

The study design of both experiments is outlined in Figure 1.

Experiment 1 was conducted over two summers (end of June–end of September) in 2018 and 2019. We chose the summer months because we suspected that the eye mask would be more helpful when dawn occurred early (as early as 05:00 am at midsummer in Cardiff, United Kingdom). The 2018 study involved two consecutive weeks in counterbalanced order, in which ambient light was blocked with an eye mask during sleep for the experimental week, and not blocked for the control week. Each week consisted of 5 habituation nights, followed by 2 consecutive testing days (Day 6 and Day 7, respectively). During the habituation nights, participants were instructed to sleep at home (wearing eye mask or control) and to maintain their regular sleep–wake habits. On Days 6 and 7, participants arrived in the sleep laboratory at Cardiff University Brain Research Centre (CUBRIC) at 08:00 am–10:00 am and performed three cognitive tasks: PAL, PVT, and MSL. The 2019 study was identical to the 2018 study, except that in the control condition, participants wore a modified eye mask with two big holes manually cut over each eye with nothing covering the eye region, such that it did not block the light. This was done in order to control any discomfort caused by wearing a mask. Computer-based tasks were executed using Matlab 2016 or 2017 (The MathWorks Inc., Natick, MA).

Experiment 2 was conducted over the summer of 2020 and consisted of four nights (two habituation and two experimental), in which participants were asked to sleep at home with the DH and an eye mask or modified mask with holes, in a counterbalanced order. Participants were given a digital light meter (Aoputtriver AP-881E, https://www.amazon.co.uk/AP-881E-Digital-Handheld-Temperature-Approved/dp/B07NVDC6CR), and instructed to place it on the pillow and to report light intensity as soon as they woke up each morning. They did this for the entire duration of the study. The first two habituation nights were used to accustom participants to sleeping while wearing both the DH and one of the masks. Between 08:00 and 10:00 am on Days 4 and 5, subjects performed the learning part of the PAL and the PVT, see Supplementary Methods for details of the tasks. Tasks were executed online using PsychoPy3 Experiment Runner (v2020.1.3 [24]). From now on, we will refer to the two conditions as: “eye mask”, for the normal sleep mask, and “control”, for both no mask (2018 participants) and the modified mask with holes over the eyes (2019/2020 participants).

In both experiments, an online sleep diary was completed every morning and self-reported alertness, was measured with the Stanford Sleepiness Scale [25], on the morning of each experimental day. Participants were asked to sleep with open shutters/curtains for the entire duration of the study.

### Wearable EEG device: Dreem headband

In Experiment 2, sleep macro-architecture was recorded using the DH that automatically records, stores, and analyses physiological data [22]. The DH consists of five dry-EEG electrodes (O1, O2, FpZ, F7, and F8). The signal is recorded with a sampling frequency of 250 Hz with a 0.4–35 Hz bandpass filter. The DH allowed participants to sleep in their own environment rather than in a laboratory setting, increasing sleep quality and comfort levels. Recent validation of the automatic sleep stage classification of the DH, compared to the standard polysomnography (PSG), showed that the automatic algorithm can reliably perform sleep staging [22].

We examined the time spent in each sleep stage during the two experimental nights (nights 3 and 4). Relationships between behavioral measures and sleep were assessed with Pearson’s correlations, or Spearman’s Rho if Shapiro–Wilk tests indicated a non-normal distribution. All statistical tests were two-tailed and considered significant at *p* < .05. Analyses were conducted in R (version 4.0.2, R Core Team, 2020). Results are presented as mean ± SEM. Four participants didn’t start the DH recording correctly, so sleep data was not collected. Sleep macrostructure analysis was, therefore, based on *N* = 29 participants.

### Behavioral analysis

We implemented a linear mixed-effects (LME) analysis in R with the *lme4* package. In all models, we included a fixed effect factor related to the type of mask (two levels: eye mask, control mask) and participant IDs as random effects. In Experiment 1 we also included a random intercept for the year of the experiment (2018, 2019): lmer (DV ~ “Mask_type” + (1|Subject) + (1|Year), data, REML = FALSE), where DV is the dependent measure. Note that we included “year” in order to capture any variability due to slight differences in the control condition in 2018 and 2019. Predictor was coded as follows: “Mask_type”: eye mask = 1, control = 0. To determine statistical significance, we conducted a likelihood ratio test (LRT) in which the full model with fixed and random effects was compared to a reduced model with random effects only. Chi-square statistic (χ^2^), associated degrees of freedom, and *p*-values for the final models are pro`vided. Additionally, Akaike Information Criteria related to a full model and to a reduced model were also reported. Significance threshold was set at 0.05. For all models, visual inspection of residual plots was used to assess the model assumptions of linearity, homoscedasticity, and normality of the residuals. Effect sizes were computed using *lsr* package [26]. All figures were created using *ggplot2* R-package [27]. Descriptive statistic of the tasks for both experiments is reported in Supplementary Results (Experiment 2), Supplementary Table S1.

All data and research materials have been made publicly available in Open Science Framework (OSF) and can be accessed at DOI 10.17605/OSF.IO/Q4P9V.

### Questionnaires

The SSS was used to provide a self-reported indication of sleepiness, with participants rating their current state on a seven-point Likert scale, where 1 is most alert and 7 is least alert [25].

A sleep diary was used to gather information about units of alcohol and caffeine consumed, sleep duration, and the regularity of the sleep–wake cycle. In Experiment 2, we also assessed the comfort of the masks and the DH on a five-point Likert scale (1: “Very uncomfortable” to 5: “Very comfortable”). Likewise, self-rating of sleep quality was measured on a Likert scale (1: “Very poor” to 5: “Very good”). Paired-sample *t*-tests were used to evaluate whether sleep quality differed after the use of the eye mask or the control. When the assumption of normality was violated, nonparametric Wilcoxon signed-rank tests were conducted instead. All tests were two-tailed and a *p*-value of <.05 was used for all analyses. Effect sizes were computed using *rcompanion* and *lsr* packages [26, 28].

## Results

### Experiment 1

#### Paired associate learning task

**Figure 2.**
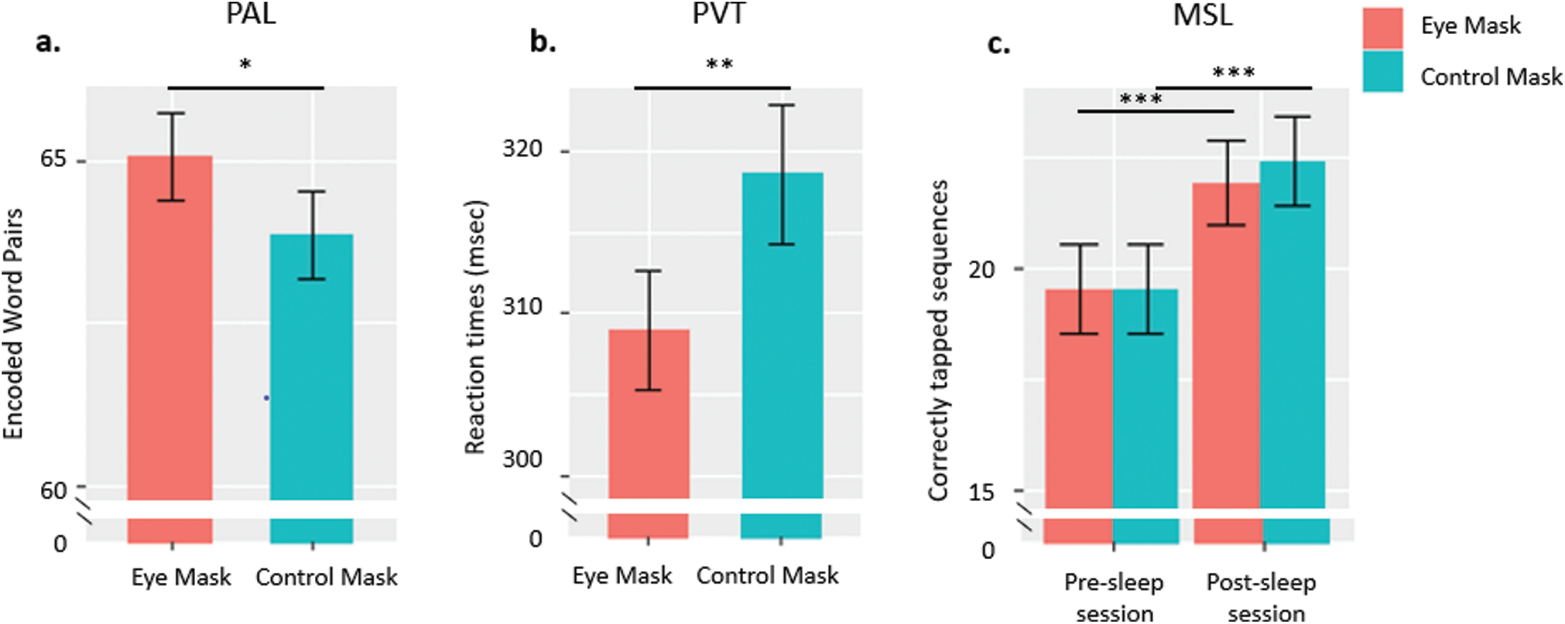
Behavioral results. Boxplots for (A) learning performance on the PAL, (B) reaction times on PVT, and (C) number of correctly tapped sequences on the MSL task. Mean +/− standard errors of the mean are indicated. *p < .05, **p < .01, ***p < .001.

We first assess whether wearing the eye mask affected learning performance on the word-pair associate task. Inclusion of “Mask_type” in our LME significantly improved model fit (χ^2^_1_ = 5.21; *p* = .022), showing significantly better learning after wearing the eye mask compared to the control (eye mask: 65.06 ± 0.69 vs control: 63.87 ± 0.67; *b* = −1.19, *p* = .023, *d* = 0.19; Figure 2a, Supplementary Table S2). An additional LME model which we fit to examine whether our experimental intervention had an impact on overnight declarative memory consolidation, revealed no effect of the eye mask on overnight change in memory performance (Supplementary Table S2).

#### Psychomotor vigilance test

We next assessed the impact of wearing the eye mask on the psychomotor vigilance task. PVT responses from the two testing days were combined in an LME which tested for differences in reaction time after wearing the eye mask as compared to the control. This revealed that blocking ambient light impacted significantly upon reaction times (χ^2^_1_ = 7.32; *p* = .006), with participants responding faster after wearing the eye mask (eye mask: 310.26 ± 2.79 vs. control: 316.37 ± 2.94; *b* = 316.37, *p* = .006, *d* = 0.16; Figure 2b, Supplementary Table S2).

#### Motor-skill learning

We examined motor-skill learning by fitting a linear mixed-effects model to the number of correctly tapped sequences with fixed effects of “Mask_type” (eye mask and control), Day (Day 6 and Day 7), and their interaction with random effects for participants and for the year of the experiment (2018 and 2019).

This showed no effect of the mask (*b* = −0.00, *p* = 1.000), but the main effect of Day (*b* = 1.19, *p* = .000), indicating that our participants performed the task faster after sleep irrespective of mask or control condition. There was no interaction between “Mask_type” and Day (*b* = 0.24, *p* = .597), indicating that our intervention did not modulate performance on this task (Figure 2c). Examination of the absolute overnight change in performance revealed no differences between the two types of masks (Supplementary Table S2).

#### Questionnaires

The LRT on the SSS performed on both testing days combined, revealed no effect of mask on self-reported alertness (χ^2^_1_ = 3.11, *p* = .078; eye mask: 2.22 ± 0.06 vs. control: 2.34 ± 0.06). Moreover, the eye mask had no impact on the actual number of hours that participants reported sleeping since a Wilcoxon signed-rank test on the total number of hours slept across the week (as indexed by the sleep diary) revealed no differences between conditions (eye mask: 8.24 ± 0.09 vs control: 8.26 ± 0.11, *Z* = −0.27, *p* = .785; *N* = 79). Notably, 10 participants were excluded from this analysis due to poor compliance in completing the sleep diary.

### Experiment 2

In Experiment 2, we sought to build on Experiment 1 by adding objective measurements of time spent in each sleep stage through the use of the DH and measurements of light intensity. However, the eye mask manipulation did not cause any changes in the sleep macrostructure, as reported in Supplementary Table S3 (see Supplementary Results (Experiment 2)) and the inclusion of light intensity in our mixed model showed no evidence that it modulated any of our behavioral measures. For consistency, we collected similar behavioral and questionnaire data as in Experiment 1. This leads to a significant result in the PAL, reported below, but no other significant findings (see Supplementary Results (Experiment 2)).

#### Paired associate learning task

**Figure 3.**
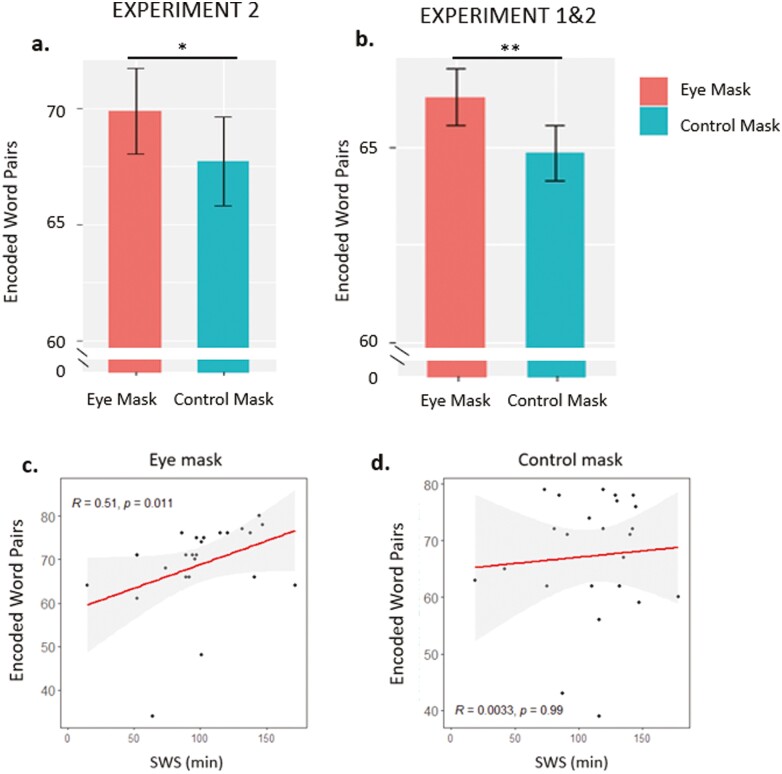
(A) Experiment 2. PAL results (*N* = 28). Boxplots for learning performance on the PAL after a night of sleep wearing the eye mask or the control mask. (B) Combined results of the encoding performance on the PAL from Experiments 1 and 2 (*N* = 112). **p < .01; *p < .05. (C) Significant Spearman’s (rank) correlation between the time spent in SWS (minutes) and the learning performance on the word pairs after a night wearing the eye mask. Note that when *N* = 3 outliers were removed, the correlation was still significant (*r*_s_ = 0.44, *p* = .04). (D) Spearman’s (rank) correlation between time spent in SWS (minutes) and learning performance on the word pairs after a night wearing the control mask.

In our assessment of mask vs control on word-pair encoding, the LRT revealed that the inclusion of “Mask_type” in the model provided a better fit for the data (χ^2^_1_ = 3.91; *p* = .047). Thus, in keeping with Experiment 1, learning performance was better after wearing the eye mask than the control (eye mask: 69.9 ± 1.89 vs control: 67.7 ± 1.80; *b* = 2.18, *p* = .049, *d* = 0.22; Figure 3a, Supplementary Table S4). Notably, this result did not change when four outliers were removed (eye mask: 72.1 ± 1.22 vs control: 69.8 ± 1.22; *b* = 2.35, *p* = .030, *d* = 0.38; Supplementary Table S4, see Supplementary Results (Experiment 2) for details).

To examine the overall dataset, we combined the PAL results related to the learning performance from both Experiment 1 and Experiment 2 (*N* = 112): lmer (DV ~ “Mask_type” + (1|Subject) + (1|Year: 2018–2019–2020), data, REML = FALSE). This indicated that the inclusion of the eye mask effect improved model fit (χ^2^_1_ = 9.01; *p* = .002). Overall, the use of the eye mask had a positive effect upon subsequent learning (eye mask: 66.6 ± 1.5 vs control: 65.1 ± 1.5; *b* = 1.44, *p* = .002, *d* = 0.19; Supplementary Table S4, Figure 3b). Given these results, we conclude that wearing the eye mask was beneficial for declarative memory encoding the next day.

In light of previous studies demonstrating that SWS plays a major role for subsequent encoding [4, 5, 7, 29], we tested for correlations between the learning of hippocampus-dependent memory and SWS time. Learning performance after a night wearing the eye mask was positively correlated with SWS time (*r*_s_ = 0.51, *p* = .011; Figure 3c), whereas there was no such correlation after a night wearing the control mask (*r*_s_ = 0.00, *p* = .99; Figure 3d). No other correlations were significant (all *p* > .05). All data and research materials have been made publicly available at the Open Science Framework (OSF) and can be accessed at DOI 10.17605/OSF.IO/Q4P9V.

#### Questionnaires

Sleep diary data revealed no differences in the number of hours slept while wearing the eye mask (7.15 ± 16.66) or the control mask (7.18 ± 16.82; *t*(23) = −0.11, *p* = .914, *N* = 24). Likewise, there was no significant difference in self-rating of sleep quality (eye mask: 3.13 ± 0.19 vs control: 2.84 ± 0.16; *Z* = −1.53, *p* = .131, *N* = 31). Participants rated the control mask as more uncomfortable than the eye mask (eye mask: 3.10 ± 0.17 vs control: 2.40 ± 0.12; *Z* = −2.86, *p* = .005, *N* = 30), but this did not impact the comfort of the DH with mask (eye mask: 2.83 ± 0.18 vs control: 2.83 ± 0.17; *Z* = 0.00, *p* = 1.000, *N* = 30).

## Discussion

Our results demonstrate that wearing an eye mask during overnight sleep can facilitate both new learning and alertness the next day.

Sleep before learning has been shown to impact subsequent encoding [4–7, 30]. For instance, Van Der Werf et al. revealed impaired declarative encoding accompanied by a decreased hippocampal activation after selective deprivation of SWS [4]. Contrary to previous studies demonstrating a reduction in the total amount of REM sleep in response to bright morning light exposure [31, 32], our examination of sleep macrostructure did not reveal any differences between a night spent wearing an eye mask and a night spent wearing a control mask. Interestingly, however, the memory improvement associated with the eye mask was positively correlated with the time spent in SWS. Encoding of declarative materials has been shown to be enhanced when slow-wave activity (SWA) is artificially increased through transcranial slow oscillation stimulation [29]. The synaptic homeostasis hypothesis posits that SWA (0.5–4 Hz), a hallmark of SWS, promotes the global down-scaling of synapses that have become saturated during preceding periods of wakefulness and thus restores capacity for the encoding of new information [33, 34]. Given this literature, we speculate that while wearing an eye mask did not increase time spent in SWS it may have increased SWA. However, we were unable to measure SWA due to the minimal nature of recording from the DH.

Turning to vigilance, the current findings suggest that wearing an eye mask has a beneficial effect upon behavioral alertness. To be specific, after a night of sleep spent wearing the eye mask, participants responded faster in the PVT. The PVT is among the most widely used measure of behavioral alertness and sustained attention, with negligible practice and aptitude effects over repeated administrations [35, 36]. A variety of studies investigating the effect of light exposure on sustained attention have used this measure after exposing participants to light treatment after a period of sleep or sleep deprivation, with the aim of counteracting sleep deprivation [37–39]. Our observation that PVT is improved after wearing an eye mask merits consideration because of the crucial role played by behavioral alertness in many real-world tasks, ranging from driving to other activities that require rapid responses [21], and because of the ecological setting in which our study was conducted. In fact, our participants slept in the comfort of their own home and were not sleep deprived; moreover, no manipulation of natural light was applied.

Turning to the motor-skill learning task, consistent with the literature, we demonstrated that participants significantly improved on this task after a retention period of sleep [40]. This overnight learning gain has previously been shown to correlate with the amount of Stage 2 sleep obtained, particularly in the last quarter of the night [40]. However, despite this overnight enhancement, the use of an eye mask does not appear to provide further benefit to this task.

Self-reported sleep quality, as assessed with the sleep diary, showed no benefits of the eye mask in either experiment. It deserves mention that even though participants in Experiment 2 reported that sleeping with the control mask was more uncomfortable in comparison with the eye mask, this did not impact self-reported sleep quality, morning alertness, or sleep parameters.

Overall, our findings suggest that a simple manipulation—the use of an eye mask during sleep—can lead to superior memory performance and higher alertness the next day. These findings have broad implications for the performance of the many daytime tasks that require learning in educational and cultural contexts, in which particularly effective encoding will determine opportunities for growth, as well as a fast response to external stimuli. Given the current climate of life-hacking, sleep monitoring, and cognitive enhancers, our findings suggest the eye mask as a simple, economical, and noninvasive way to get more out of a night of sleep.

## Supplementary Material

zsac305_suppl_Supplementary_Material

## Data Availability

All data and research materials have been made publicly available in Open Science Framework (OSF) and can be accessed at DOI 10.17605/OSF.IO/Q4P9V.

## References

[1] Killgore WDS. Effects of Sleep Deprivation on Cognition. Prog Brain Res. 2010;185:105–129. doi:10.1016/B978-0-444-53702-7.00007-5. 21075236

[2] Diekelmann S , et al. The memory function of sleep. Nat Rev Neurosci. 2010;11(2):114–126. doi:10.1038/nrn2762. 20046194

[3] Lange T , et al. Effects of sleep and circadian rhythm on the human immune system. Ann N Y Acad Sci. 2010;1193(1):48–59. doi:10.1111/j.1749-6632.2009.05300.x. 20398008

[4] van der Werf YD , et al. Sleep benefits subsequent hippocampal functioning. Nat Neurosci. 2009;12(2):122–123. doi:10.1038/nn.2253. 19151712

[5] Yoo SS , et al. A deficit in the ability to form new human memories without sleep. Nat Neurosci. 2007;10(3):385–392. doi:10.1038/nn1851. 17293859

[6] Cousins JN , et al. Memory encoding is impaired after multiple nights of partial sleep restriction. J Sleep Res. 2018;27(1):138–145. doi:10.1111/jsr.12578. 28677325

[7] van der Werf YD , et al. Reduction of nocturnal slow-wave activity affects daytime vigilance lapses and memory encoding but not reaction time or implicit learning. Prog Brain Res. 2011;193:245–255. doi:10.1016/B978-0-444-53839-0.00016-8. 21854967

[8] Curcio G , et al. Sleep loss, learning capacity and academic performance. Sleep Med Rev. 2006;10(5):323–337. doi:10.1016/j.smrv.2005.11.001. 16564189

[9] Daan S , et al. Timing of human sleep: recovery process gated by a circadian pacemaker. Am J Physiol Regul Integr Comp Physiol. 1984;15(2):161–183. doi:10.1152/ajpregu.1984.246.2.r161. 6696142

[10] Wams EJ , et al. Linking light exposure and subsequent sleep: a field polysomnography study in humans. Sleep. 2017;40(12):1–13. doi:10.1093/sleep/zsx165. PMC5806586 29040758

[11] Blume C , et al. Effects of light on human circadian rhythms, sleep and mood. Somnologie. 2019;23(3):147–156. doi:10.1007/s11818-019-00215-x. 31534436 PMC6751071

[12] Schmidt TM , et al. Intrinsically photosensitive retinal ganglion cells: many subtypes, diverse functions. Trends Neurosci. 2011;34(11):572–580. doi:10.1016/j.tins.2011.07.001. 21816493 PMC3200463

[13] Borbely AA. A two process model of sleep regulation. Hum Neurobiol. 1982;1(3):195–204. 7185792

[14] Borbély AA , et al. The two-process model of sleep regulation: a reappraisal. J Sleep Res. 2016;25(2):131–143. doi:10.1111/jsr.12371. 26762182

[15] Badia P , et al. Bright light effects on body temperature, alertness, EEG and behavior. Physiol Behav. 1991;50(3):583–588. doi:10.1016/0031-9384(91)90549-4. 1801013

[16] Dijk DJ , et al. Light, sleep, and circadian rhythms: together again. PLoS Biol. 2009;7(6):e10001457–e10001410. doi:10.1371/journal.pbio.1000145. PMC2691600 19547745

[17] Wunderlin M , et al. Modulating overnight memory consolidation by acoustic stimulation during slow-wave sleep: a systematic review and meta-analysis. Sleep. 2021;44(7):1–11. doi:10.1093/sleep/zsaa296. 33406249

[18] Bani Younis M , et al. Effectiveness of using eye mask and earplugs on sleep length and quality among intensive care patients: a quasi-experimental study. Int J Nurs Pract. 2019;25(3):1–9. doi:10.1111/ijn.12740. 31090172

[19] Locihová H , et al. Effect of the use of earplugs and eye mask on the quality of sleep in intensive care patients: a systematic review. J Sleep Res. 2018;27(3):1–12. doi:10.1111/jsr.12607. 28944590

[20] Anderson C , et al. PVT lapses differ according to eyes open, closed, or looking away. Sleep. 2010;33(2):197–204. doi:10.1093/sleep/33.2.197. 20175403 PMC2817906

[21] Dorrian J , et al. Psychomotor vigilance performance: neurocognitive assay sensitive to sleep loss. In: Kushida CA, ed. Sleep Deprivation: Clincal Issues, Pharmacology, and Sleep Loss Effects; New York, NY, USA: Marcel Dkker, Inc. 2005; pp. 39–70. doi:10.3109/9780203998007-4.

[22] Arnal PJ , et al. The dreem headband compared to polysomnography for electroencephalographic signal acquisition and sleep staging. Sleep. 2020;43(11):1–13. doi:10.1093/sleep/zsaa097. PMC7751170 32433768

[23] Faul F , et al. Statistical power analyzes using G*Power 3.1: tests for correlation and regression analyses. Behav Res Methods. 2009;41(4):1149–1160. doi:10.3758/BRM.41.4.1149. 19897823

[24] Peirce J , et al. PsychoPy2: experiments in behavior made easy. Behav Res Methods. 2019; 51(1):195–203. doi:10.3758/s13428-018-01193-y. 30734206 PMC6420413

[25] Hoddes E , et al. Quantification of sleepiness: a new approach. Psychophysiology. 1973;10(4):431–436. doi:10.1111/j.1469-8986.1973.tb00801.x. 4719486

[26] Navarro DJ. Learning Statistics with R: A Tutorial for Psychology Students and Other Beginners (Version 0.6). Danielle Navarro University of New South Wales; 2015: 1–564. https://learningstatisticswithr.com/

[27] Wickham H. Ggplot2 elegant graphics for data analysis. Media. 2009;35(July):211.

[28] Mangiafico SS. *rcompanion: Functions to Support Extension Education Program Evaluation* [R Statistical Package]. 2019; (September 2016). http://rcompanion.org/

[29] Antonenko D , et al. Napping to renew learning capacity: enhanced encoding after stimulation of sleep slow oscillations. *Eur J Neurosci.* 2013;37(7):1142–1151. doi:10.1111/ejn.12118. 23301831

[30] McDermott CM , et al. Sleep deprivation causes behavioral, synaptic, and membrane excitability alterations in hippocampal neurons. J Neurosci. 2003;23(29):9687–9695. doi:10.1523/jneurosci.23-29-09687.2003. 14573548 PMC6740462

[31] Dijk DJ , et al. Bright morning light advances the human circadian system without affecting NREM sleep homeostasis. Am J Physiol Regul Integr Comp Physiol. 1989;256(1):106–111. doi:10.1152/ajpregu.1989.256.1.r106. 2912203

[32] Dijk DJ , et al. Reduction of human sleep duration after bright light exposure in the morning. Neurosci Lett. 1987;73(2):181–186. doi:10.1016/0304-3940(87)90014-0. 3822250

[33] Tononi G , et al. Sleep and synaptic homeostasis: a hypothesis. Brain Res Bull. 2003;62(2):143–150. doi:10.1016/j.brainresbull.2003.09.004. 14638388

[34] Tononi G , et al. Sleep and the price of plasticity: from synaptic and cellular homeostasis to memory consolidation and integration. Neuron. 2014;81(1):12–34. doi:10.1016/j.neuron.2013.12.025. 24411729 PMC3921176

[35] Lim J , et al. Sleep deprivation and vigilant attention. Ann N Y Acad Sci. 2008;1129:305–322. doi:10.1196/annals.1417.002. 18591490

[36] Basner M , et al. Maximizing sensitivity of the Psychomotor Vigilance Test (PVT) to sleep loss. Sleep. 2011;34(5):581–591. doi:10.1093/sleep/34.5.581. 21532951 PMC3079937

[37] Phipps-Nelson J , et al. Daytime exposure to bright light, as compared to dim light, decreases sleepiness and improves psychomotor vigilance performance. Sleep. 2003;26(6):695–700. doi:10.1093/sleep/26.6.695. 14572122

[38] Münch M , et al. Blue-enriched morning light as a countermeasure to light at the wrong time: effects on cognition, sleepiness, sleep, and circadian phase. Neuropsychobiology. 2017;74(4):207–218. doi:10.1159/000477093. 28637029

[39] Comtet H , et al. Light therapy with boxes or glasses to counteract effects of acute sleep deprivation. Sci Rep. 2019;9(1):1–9. doi:10.1038/s41598-019-54311-x. 31792259 PMC6889287

[40] Walker MP , et al. Practice with sleep makes perfect: sleep-dependent motor skill learning. Neuron. 2002;35(1):205–211. doi:10.1016/s0896-6273(02)00746-8. 12123620

